# How do the incomes of Israeli Arab physicians compare with those of Israeli Jewish physicians?

**DOI:** 10.1186/s13584-026-00769-w

**Published:** 2026-06-24

**Authors:** Bruce Rosen, Sami Miaari

**Affiliations:** 1 Myers-JDC-Brookdale Institute, Jerusalem, Israel; 2 The Hebrew University, Jerusalem, Israel; 3 Tel Aviv University, Tel Aviv, Israel; 4 Yale University, New Haven, Connecticut, USA

## Abstract

**Background:**

In 2023 Israeli Arabs (i.e., Arab Palestinian citizens of Israel) constituted 22% of Israel’s working-age population. In that same year, Israeli Arabs constituted 25% of Israel’s employed physicians of working age - up significantly from 8% in 2010. The objectives of this study were to: 1) assess whether, and to what extent, there is an Arab-Jewish income differential among Israeli physicians; 2) assess the extent to which any such differential can be attributed to Arab-Jewish differences in the demographic, geographic, and/or work-related characteristics of the medical workforce; and 3) explore the policy implications of the key findings.

**Methods:**

The analysis utilized the Central Bureau of Statistics' 2022 Population and Housing Census, which included data on 7,089 physicians, of whom 1,333 were Arab and 5,756 were Jewish or other (hereafter “Jewish”). The main income variable examined was “Total annual gross income from work”.

**Results:**

In 2022, the average annual physician income for Arab physicians was 26% less than for Jewish physicians (NIS 358 thousand vs. NIS 483 thousand). Arab physicians were more likely to be under age 40 (71% v. 28%), male (77% v. 52%), residents of the North region (48% v. 7%), in non-supervisory roles (87% v. 75%), and generalists or family practitioners (33% v. 22%). Controlling for age and sex, via regression analysis, reduced the income differential to 1%. This major reduction was due to Arab physicians being markedly younger than Jewish physicians. Further controlling for regional distribution and work characteristics (managerial status, specialty status/type, and months worked) did not change the ethnicity differential, but did markedly reduce the coefficients of the age variables.

**Conclusions:**

The 2022 Arab-Jewish income differential among physicians was due predominantly to differences in age composition. The differential could potentially shrink in the decades ahead, as more Arab physicians reach the ages at which physician incomes are at their highest. However, as part of the effect of age on income is mediated by work characteristics, the extent to which the wage gap will shrink will depend in part on the extent to which Arab physicians will secure prestigious residency training slots and managerial positions. Health system leaders can play an important role in promoting such developments. In particular, steps should be taken to increase the representativeness of Arabs in Israeli medical schools.

## Background

Wage disparities by race and ethnicity are well-documented globally. To some extent, these wage disparities have been attributed to differences in education and other factors that could influence productivity [1]. However, studies from the USA and England reveal persistent income gaps that are not entirely explained by education, work experience, or hours worked [1–4]. Such gaps have also been documented globally in a variety of high-skill professions [4].

Similar patterns obtain in Israel, where Israeli Arabs (Arab Palestinian citizens of Israel) constitute approximately one-fifth of the population. Arab-Jewish wage disparities in Israel are well-documented [5] and they reflect broader ethnic inequalities [6]. Studies have shown that Arabs are systematically underrepresented in high-paying positions and overrepresented in low-skill or manual labor sectors [5].

Until recently, Israeli Arabs also lagged behind in educational levels [7], but over the past decade the differences in education levels have narrowed, with the number of Israeli Arabs enrolled in institutions of higher education more than doubling [8]. Yet even among highly educated Arabs, employment outcomes have lagged behind those of Jews, reflecting barriers such as residential segregation, discrimination, and differential returns to education [6, 9].

To what extent have economy-wide wage disparities between minority and majority populations been reflected the health care professions – both globally and in Israel?

Globally the healthcare sector plays a pivotal role in fostering equity, inclusion, and access to essential services. However, in many countries, disparities persist in the treatment and compensation of minority healthcare professionals. In the United States, for instance, Black and Hispanic physicians earn less than their white counterparts even after accounting for specialty and hours worked [10]. This gap partly stems from minority physicians’ higher likelihood of working in underserved areas and having fewer opportunities for leadership roles [11]. Gender and ethnicity intersect to amplify disparities, as seen in studies within the NHS in England, where Asian and Black female doctors earn significantly less than their male counterparts [12].

The lower incomes of minority professionals probably reflect, in part, broader societal inequities, such as differences in the quality of elementary and secondary education, unequal access to prestigious training institutions and discrimination in hiring and promotion [4]. These patterns underscore the importance of understanding how racial or ethnic background influences earnings in specific sectors like healthcare [10–12].

In Israel, the extent and nature of Arab-Jewish wage differentials in the health professions have not been studied systematically. However, a recent article by the authors [13] has laid important groundwork for such a study. We found that Israeli Arabs constitute a substantial and growing percentage of Israeli health care professionals in the fields of medicine, nursing, dentistry and pharmacy. In 2023, their share in employment in these four professions ranged from 25% to 50% - all substantially greater than the share of Israeli Arabs in the employment in all the academic professions taken together (9%). With regard to physicians, we found that Arab representation increased from 8% in 2010 to 25% in 2023.^1^

We also explored the factors that contributed to these developments. These included the universal and humanistic values of health care, the public financing of Israeli health care, supply shortfalls in some of the health professions, and the willingness of Israeli Arabs to study in universities outside of Israel.

However, while our recent paper examined data on professional studies, licensure, and employment, it did not include an examination of income levels. This left open the question of whether there are substantial Arab-Jewish income gaps in the health professions. Moreover, findings from our previous study had suggested that, despite the substantial and increasing representation of Israeli Arabs in medicine, there were still reasons to be concerned about income differentials. For example, Arab physicians often studied abroad^2^, a factor that limits their access to competitive residencies and leadership positions in Israel. This dynamic aligns with findings from other sectors, where Arabs face structural barriers to managerial and senior professional roles [5].

The main reason that our previous paper did not examine income disparities was that, at the time, recent data on income were not available for a large enough sample. That situation changed in December 2024, when the Central Bureau of Statistics (CBS) made micro-level data from its 2022 Population and Housing Census available in the CBS research rooms. The newly available database made it possible to explore in-depth the magnitude and nature of Arab-Jewish income disparities among physicians in Israel, a critical yet unstudied issue.

The objectives of this study were to: (1) Assess whether, and to what extent, there is an Arab-Jewish income differential among Israeli physicians; (2) Assess the extent to which any Arab-Jewish income differential among physicians can be attributed to Arab-Jewish differences in the sociodemographic, locational, and/or work-related characteristics of the medical workforce; (3) Explore the policy implications of the key findings.

We hypothesized that the incomes of Arab physicians would be lower than those of Jewish physicians. The reasons for this hypothesis were that (a) Arabs are more likely to have studied medicine outside of Israel, and the Israeli medical system puts a premium on study in Israel, and (b) In many sectors of the Israeli economy, Arabs are under-represented in managerial positions and senior professional positions.

## Methods

### The data source

The analysis is based on data from the 2022 Census of the Central Bureau of Statistics, which includes detailed demographic and occupational information on 7,089 physicians, of whom 1,333 were Arab and 5,756 were Jewish or other (hereafter “Jewish”).^3^ The census data is uniquely suited for this study as it provides reliable income information for a variety of income measures, alongside sociodemographic and work-related characteristics. It also has the advantage of including enough Arab physicians for a detailed comparison of Arab-Jewish income differentials. The census is considered to be a very reliable data source which is representative of the Israeli population.

The CBS census datafile includes both administrative data and data from a survey of a large representative sample of Israeli households. For example, data on income come from an administrative source (the Israel Tax Authority), while data on occupation come from the responses to the survey.

### The key variables

Our main dependent variable was total gross annual income from work. In the findings section of the main text, we present a series of means comparisons, crosstabs and OLS regressions for this dependent variable^4^.

The main groups of independent variables were as follows:

- **Ethnicity**: A binary indicator for Arab vs. Jewish.
- **Demographic characteristics**: Age (categorized into groups) and sex.
- **Location**: District of residence.
- **Work-related characteristics**: Supervisory role (supervisory vs. non-supervisory), specialty status (resident, generalist or family physician, other specialist), months worked in the past year.

Note that all statistics presented in this manuscript include both Arab Palestinian citizens of Israel and Palestinians from East Jerusalem. They do not include Palestinians from the West Bank and Gaza.^5^ In addition, in keeping with standard Central Bureau of Statistics (CBS) practice, the variable “population group” is divided into two groups: “Arabs” and “Jews and others”. As the latter group is predominantly composed of Jews, we refer to it as “Jews” throughout this manuscript.

### The analysis plan

Crosstabulations and comparisons of means were utilized to provide a descriptive analysis of income disparities. These methods allow the identification of how Jewish and Arab physicians differ with regard to key background variables, as well as the patterns in income differences by ethnicity, age, sex, location, and workplace characteristics.

Observations were weighted to reflect sampling likelihood, using the weights specified by the Central Bureau of Statistics.

Regression analyses were used to assess the extent of Arab-Jewish income differentials, with independent variables added sequentially – as follows:

- Model 1: ethnicity.
- Model 2: sex.
- Model 3: age.
- Model 4: region.
- Model 5: work characteristics.

In examining these models, our main interest was in how the coefficient of the ethnicity variable changed as each group of other variables were added (with only a secondary interest in the coefficients of the other variables).

The analysis gives particular attention to models 3 and 5. We refer to model 3 as “the basic model” as it is the fullest model to include only inherent characteristics of the physicians (ethnicity, sex, and age) which are determined from birth and hence exogenous to the health system. The unique contribution of the basic model is that it estimates the average pay differential between Arab and Jewish physicians with the same inherent characteristics.

We refer to model 5 as “the expanded model” as it is the fullest model as it also includes variables endogenous to the health system (particularly work characteristics). The unique contribution of the enhanced model is that it estimates the average pay differential between Arabs and Jews with the same inherent characteristics for the same type of job, location, and months worked.

Taken together, the basic and expanded models provide information on the extent to which Arab-Jewish pay differentials adjusted for age and sex can be attributed to differences in location and job characteristics.

### The plan for supplementary analyses presented in appendices

The analyses presented in the main text used total income from work as the dependent variable. Analyses were also carried out separately for income from salaried work and income from independent work; these are presented in Appendix A.

In the interest of simplicity, the regressions presented in the main text do not include any interaction terms (such as age-ethnicity variables). This has the limitation of not giving expression of possible differences between Arabs and Jews in the relationships between income and the demographic, location, and workplace characteristics. Appendix B addresses this limitation by presenting regressions with interaction terms as well as split sample regression results.

Another limitation of the standard OLS regressions is that they relate only to the average Arab-Jewish income differential. This is addressed in Appendix C which compares the income deciles for Arabs and Jews and presents the results of quantile regressions.

The use of gross income in the regressions has the advantage of yielding findings which can be readily understood by non-economists. However, economists note that gross income variables rarely have a normal distribution, and hence they prefer regressions using the natural logarithm of gross income as the dependent variable. In Appendix D, we present regressions using the natural logarithm of income.

Appendix E presents additional information from analyses in which ethnicity and sex are considered conjointly.

Appendix F explores the extent to which the incomes of Israeli Arab physicians may be benefitting from starting medical careers at a relatively young age.

Appendix G examines Arab-Jewish income differentials across a variety of professions, thereby providing context for the findings regarding physicians.

Appendix H compares our findings from the 2022 Census with findings from the 2008 census.

## Results

In 2022, the average annual physician income in Israel was NIS 454 thousand; it was NIS 483 thousand for Jews and NIS 358 thousand for Arabs. Thus, on average, Arab physicians earned 26% less than Jewish physicians^6^.

As indicated in Table 1, the Arab and Jewish physicians also differed markedly in their age and sex compositions. The Arab physicians tended to be significantly younger (with 71% below the age of 40, compared with 28% among Jewish physicians). Also, the proportion of the physicians who were male was much higher among Arabs (77%) than among Jews (52%).

**Table 1:** The age and sex composition of the medical workforce, by ethnicity (percents)

	Total	Arabs	Jews
Age group			
Up to 29	8	26	3
30 to 39	29	45	25
40 to 49	17	11	19
50 to 59	18	8	21
60 to 69	19	10	22
70 and over	9	1	11
Sex			
Male	58	77	52
Female	42	23	48
N	28,112	8,432	36,544

**Fig. 1:**
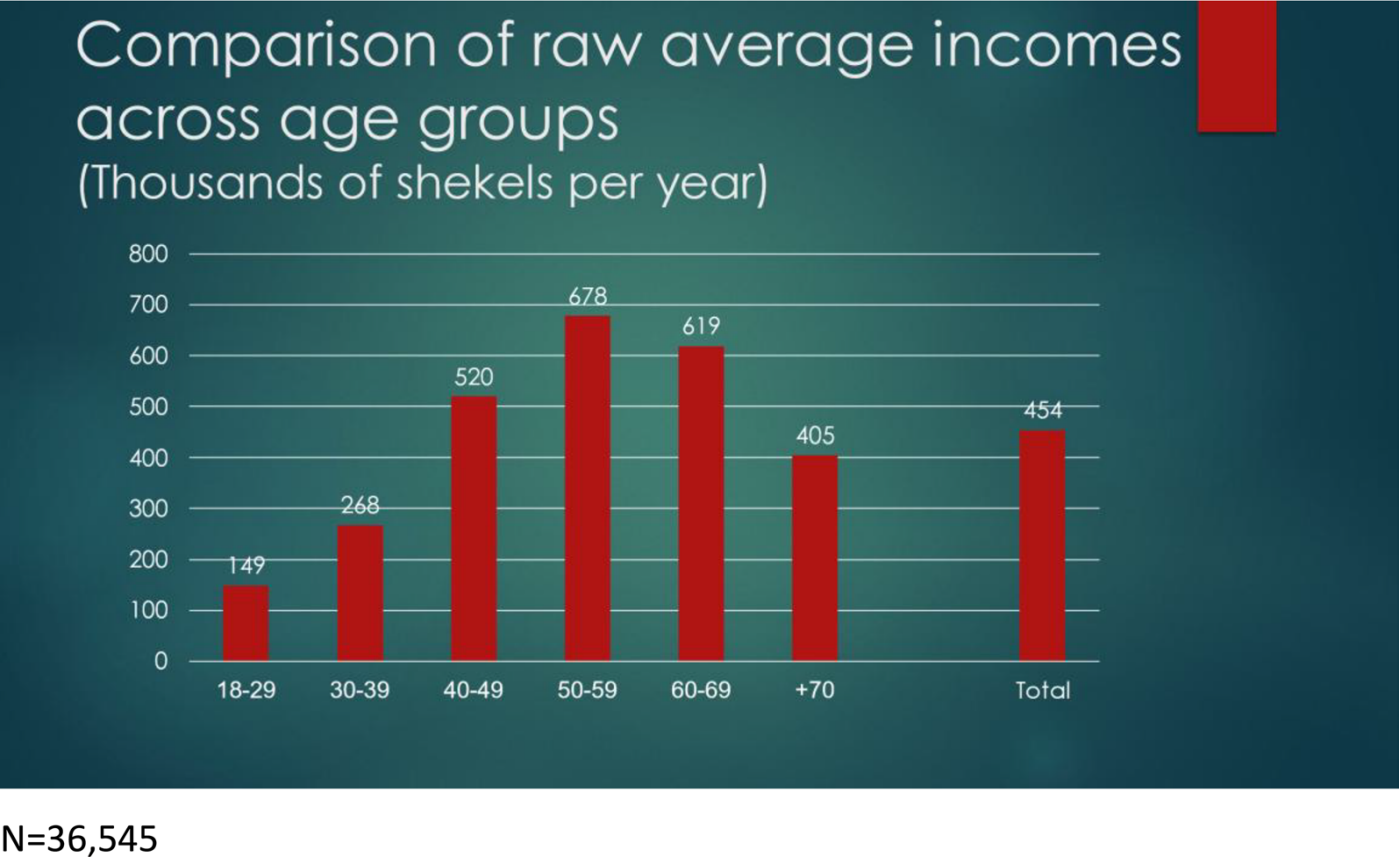
Comparison of raw and average incomes across age groups

There were strong associations between physician income and both sex and age. The average income was NIS 507 thousand for male physicians and NIS 382 thousand for female physicians. As indicated in fig. 1, physician incomes increased sharply with age until peaking with the 50–59 age group, then declined for the older age groups.

**Fig. 2:**
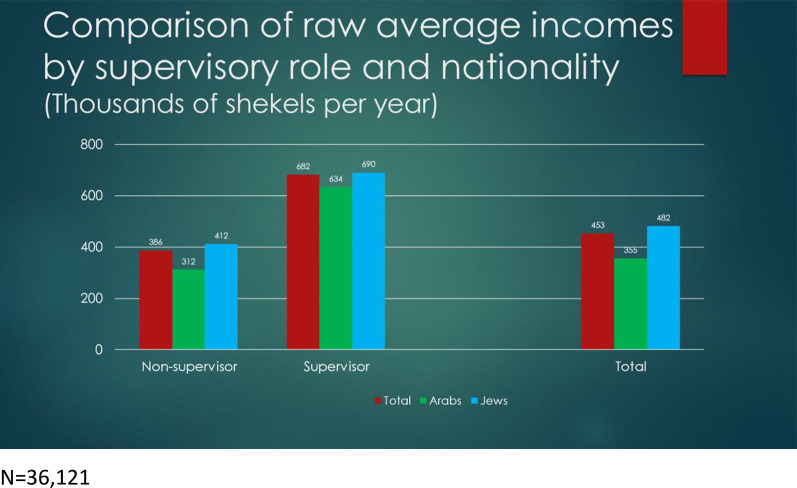
Comparison of raw average incomes by supervisor role and nationality

In addition to the demographic differences between Arab and Jewish physicians, the two groups also differed in terms of several income-relevant work characteristics (Table 2). For example, Jewish physicians were almost twice as likely as Arab physicians to be in supervisory roles. As can be seen in fig. 2, physicians in supervisory roles tended to earn substantially more than physicians in non-supervisory roles. Significantly, among physicians in supervisory roles, average incomes for Arabs were almost as high as for Jews.

**Fig. 3:**
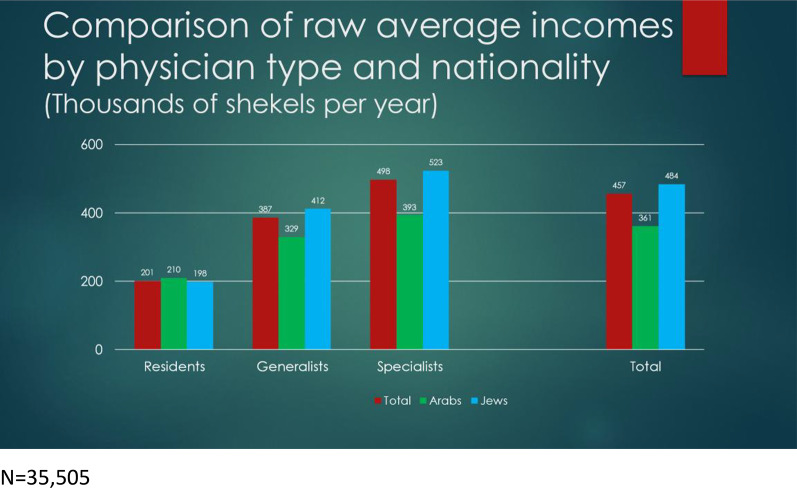
Comparison of raw average incomes by physician type and nationality

Table 2 also indicates that Jewish physicians were more likely than Arab physicians to be board-certified specialists in fields other than family medicine. As indicated in fig. 3, those specialists earned approximately 25% more, on average, than other non-resident physicians (generalists along with board certified family physicians) – a category more prevalent among Arab physicians.

Another relevant income-related work characteristic (not shown in Table 2) is the number of months worked over the past year. This averaged about 5% higher for Jewish physicians compared with Arab physicians − 11.5 months v 11.1 months.

**Table 2:** Distributions of selected work characteristics, by ethnicity (percents)

	Total	Arabs	Jews
Hierarchical roles (*N* = 36,122)			
Supervisory	23	13	25
Non-supervisory	77	87	75
Specialty status (*N* = 35,505)			
Resident	5	6	5
Generalist or family physician	24	33	22
Other specialists	71	61	73

**Fig. 4:**
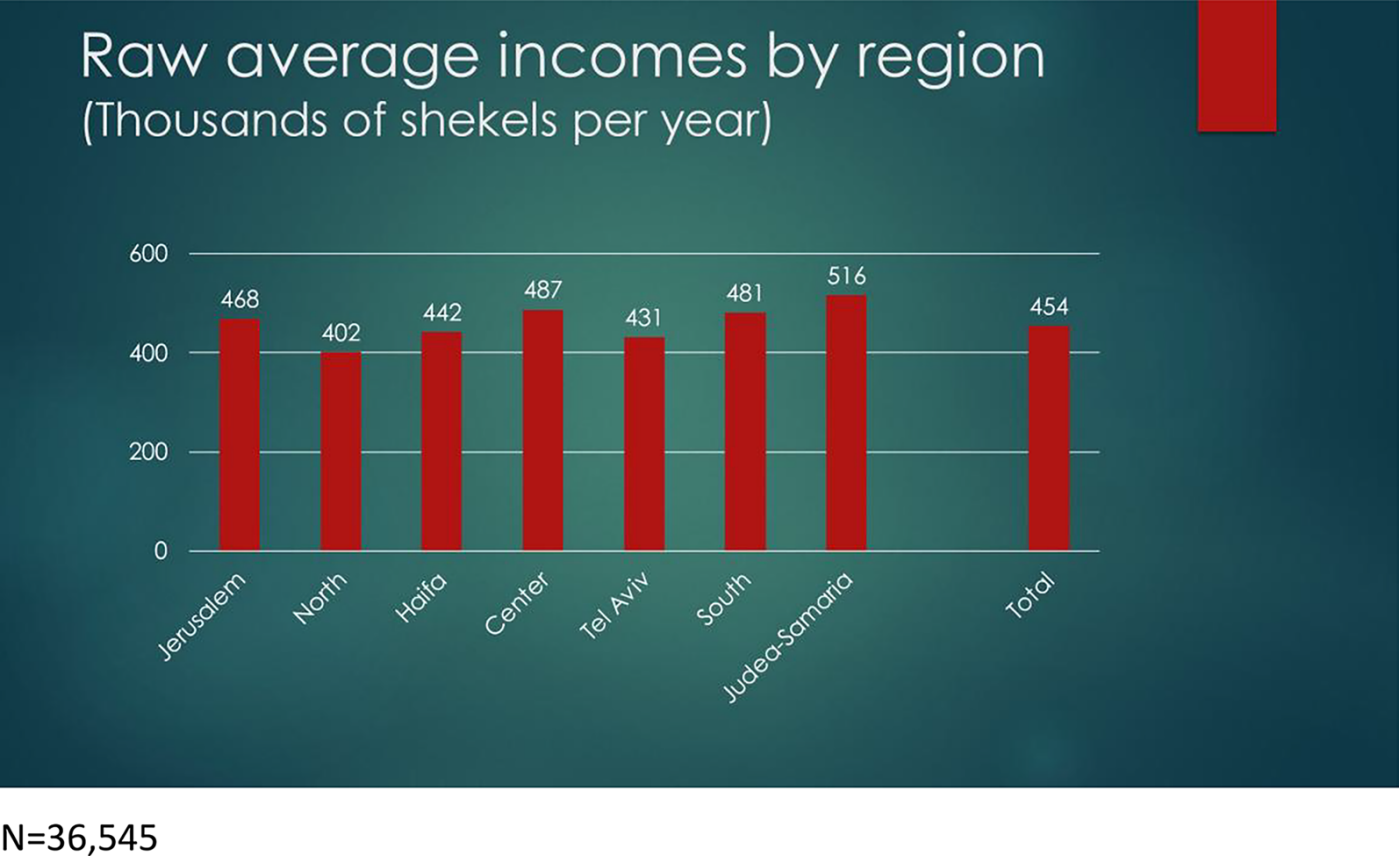
Raw average incomes by region

As indicated in fig. 4, there were also substantial differences in physician incomes across regions, with the North as the region with the lowest average income.^7^ As indicated in Table 3, the physicians in the North were also characterized bya much higher concentration of Arabs^8^, a higher concentration of physicians under age 40, and lower concentrations of females, specialists (aside from those in family medicine), and supervisors.^9^

Note also that 48% of Arab physicians resided in the North, while only 7% of Jewish physicians resided in that region.

**Table 3:** Selected physician characteristics - North v other regions (percents)

	Total	North	Other
Arabs	23	68	14
Females	42	33	44
Age under age 40	37	54	34
Specialists (other than family practice)	71	64	72
Supervisors	23	17	24
N	36,545	5,935	30,610

Table 4 presents the results of a series of regressions, all of which used “total annual income from work” as the dependent variable. When ethnicity was the only independent variable (model 1), its coefficient (−124,817) was negative, large, and statistically significant. The addition of sex as an independent variable (model 2) slightly increased the magnitude of the negative coefficient of the ethnicity variable, while the subsequent addition of age as an independent variable (model 3) markedly reduced the magnitude of that coefficient and rendered it statistically non-significant^10^. Controlling also for region (model 4) slightly increased the magnitude of the negative coefficient for ethnicity. Controlling for the work characteristics of managerial status, specialty status, and months worked (model 5) reduced the magnitude of the negative ethnicity coefficient, which remained not statistically significant. The addition of the work characteristics also reduced the magnitude of the age coefficients.

Note that in model 1, where ethnicity is the only independent variable, its coefficient is significant, but the model explained only 2% of the variation in total income. The addition of sex in model 2 increased the R-squared to 0.06 and increased the negative coefficient of the ethnicity variable. The addition of age variables in model 3 substantially increased the extent to which the variation in income was explained – to 25% - and rendered the ethnicity variable non-significant. The addition of a regional dummy variables in model 4 did not increase the extent to which the variation in income was explained and barely changed the coefficient of the age, and sex variables; however, it did increase the magnitude of the negative coefficient of the ethnicity variable and rendered it statistically significant. The addition of work characteristics in model 5 increased the extent of variation explained to 30%, decreased the magnitude of the negative coefficient of the ethnicity variable to a level similar to that of model 3, and reduced the coefficients of the age variables. This implies that the effect of age on income is, in part, mediated by work characteristics (supervisory role and specialty status/type).

The inclusion of work characteristics in Model 5 led to several changes that are suggestive regarding the underlying mechanism: (a) attenuation of the age coefficients, (b) a substantial increase in the R-squared, (c) an R-squared which remains well below 100%. This is consistent with age affecting earnings in part through observable career-related roles, while also leaving open the possibility of a significant role for unobserved variables effecting pay – both directly and as additional mediators of age, per the Oster framework [14].

The implications of these regressions for the Arab-Jewish income differential among physicians are summarized in Table 5. It indicates that in terms of the raw data, the Arab-Jewish income differential was 26% of the average Jewish income. Controlling for inherent demographic characteristics (particularly age) reduced this differential to 1%^11^. Controlling for both location and work characteristics did not change the magnitude of that differential.

**Table 4:** Regression results for total income

	Model 1	Model 2	Model 3	Model 4	Model 5
R-squared	0.02	0.06	0.25	0.25	0.31
Constant	482,591	55,744	208,214	223,362	239,534
Ethnicity (Arab)	**−124**,**817**	**−164**,**362**	−5,413	**−12**,**013**	−5,335
Sex (Female)		**−155**,**334**	**−126**,**857**	**−126**,**529**	**−111**,**825**
Age 30–39			**119**,**073**	**120**,**299**	**81**,**890**
Age 40–49			**373**,**128**	**372**,**957**	**274**,**195**
Age 50–59			**527**,**712**	**526**,**842**	**417**,**604** **30**,**299**
Age 60–69			**460**,**549**	**459**,**843**	**358**,**339**
Age 70 +			**230**,**736**	**229**,**716**	**159**,**707**
North				**−20**,**310**	**−28**,**024**
Haifa				**−26**,**265**	**−25**,**258**
Center				**−23**,**990**	**−33**,**470**
Tel Aviv				**−39**,**761**	**−52**,**106**
South				**−19**,**350**	−9,639
Judea & Samaria				−22,780	−20,579
Non-supervisor					**−154**,**111**
Resident					14,818
Specialist (not FP)					**87**,**162**
Months worked					**24**,**286**
N	36,544	36,544	36,544	36,544	34,897

Coefficients in bold are significant at the 0.05 level

**Table 5:** How does controlling for demographic and work characteristics affect the Arab-Jewish income differential?

	**Absolute differential**(Thousands of shekels per year)	**Relative differential**(Relative to average Jewish physician income)
**Simple model**(Ethnicity as sole independent variable)	-124	-26%
**Basic model**(After adding demographic variables)	-5	-1%
**Intermediate model**(After also adding region variables)	-12	-2%
**Enhanced model**(After also adding work variables)	-5	-1%

**Fig. 5:**
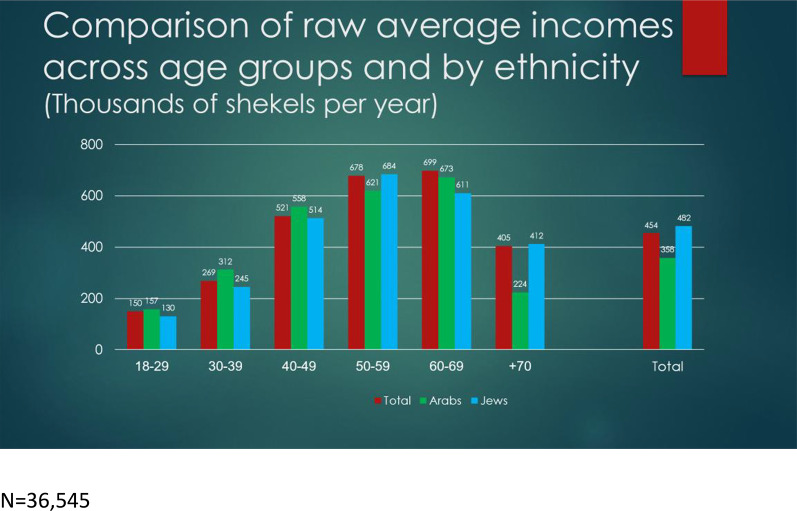
Comparison of raw average incomes across age groups and by ethnicity

Considering the significant role of age in the regressions, it is noteworthy that, within age groups, the Arab-Jewish income differentials tended to be relatively small, aside from the 70 + age group (fig. 5).

**Fig. 6:**
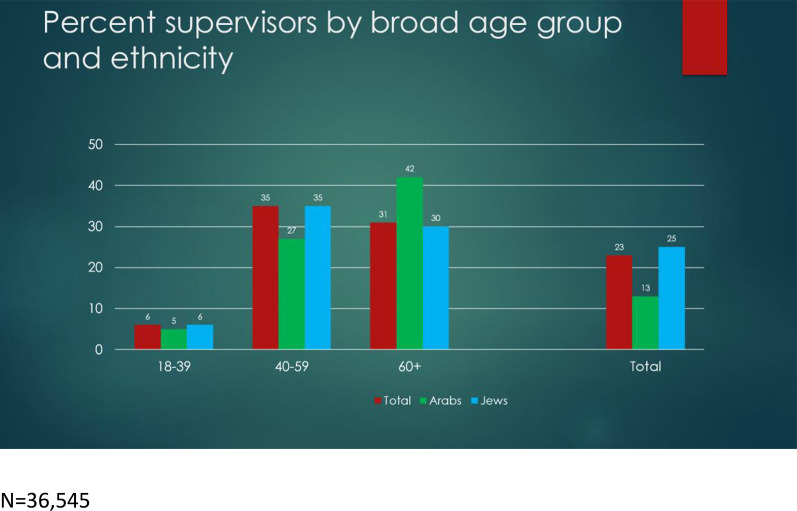
Percent supervisors by broad age group and ethnicity

In light of the regression analysis, it is important to note that the prevalence of supervisory roles – among both Jews and Arabs - is substantially higher among physicians over age 40 than among younger physicians (fig. 6). At the same time, among physicians aged 40–49 the prevalence of supervisory roles is markedly greater among Jewish physicians than among Arab physicians. The reverse is true among physicians aged 60–68.

### Summary of additional analyses presented in the appendices

In Israel, both salaried work and independent work contribute significantly to physician income. Appendix A presents an analysis which distinguishes between those two types of incomes – both for all physicians taken together and separately for Arab and Jewish physicians. It shows that independent income constituted a substantially larger percentage of total income for Jewish physicians compared with Arab physicians (25% v 16%). It also shows that, for independent income, there was a substantial Arab-Jewish raw income differential (53%), which was about triple the raw differential for salaried income (17%). However, even for independent income the Arab-Jewish income differential narrow markedly (to approximately 5%) after controlling for age, sex, region and work characteristics. Finally, while the regression for salaried income accounts for 25% of the variance in the dependent variable, the parallel figure for independent income was only 9%.

Appendix B presents the findings of regressions in which the dependent variable was the natural logarithm of income rather that income. The story that emerges there is very similar to the story presented above for income, per se.

Appendix C presents the findings of split sample regression for Model 5, run separately for Arabs and Jews, as well as a regression using interaction terms. A key finding from the split sample analysis was that increases in age have a greater positive effect among Arab physicians than among Jewish physicians, with the 50–59 age group constituting a notable exception. However, in the regression analyses the age-ethnicity variables had a mix of positive and negative coefficients.

Appendix D compares the income distributions separately for Arab physicians and Jewish physicians as well as quantile regressions. A key finding from the comparison of distributions was that, with increases in income deciles, the absolute differential increased, while the percentile differential decreased. A key finding from the quantile regressions was that, within income quartiles, after controlling for age and sex, incomes tended to be higher for Arab physicians than for Jewish physicians.

Appendix E compares Arab and Jewish physician incomes by sex. A key finding was that the Arab-Jewish income differential was greater among female physicians than among male physicians. Another key finding was that the male-female income differential was greater among Arabs than among Jews. Analyses presented in this appendix and in Appendix B suggest that both of these findings may be due to the relatively recent expansion in the number of Arab female physicians and hence their concentration in the younger (and lower-paying) age groups.

Appendix F explores the extent to which the similarity in Arab-Jewish physician incomes (particularly after controlling for age) might be due in part to a tendency of Arabs to start medical school at a relatively younger age (due to exemption from IDF service), as this might translate into more years of experience at any given age, and hence more income at any given age. The analysis suggests that such an “experience effect” could be non-negligible, but is not predominant.

Appendix G provides context for the Arab-Jewish income differentials for physicians by providing comparable data for several additional professions: nursing, dentistry, pharmacy, law, and teaching. It shows that the Arab-Jewish income differentials were lower in the professions which work primarily in the public sector (medicine, nursing, and teaching) than in those which work primarily in the private sector (dentistry and law).

Appendix H compares our findings from the 2022 census with parallel findings from the 2008 census (i.e. the most recent census prior to 2022). In both years, the raw income differential between Arab and Jewish physicians was approximately 25%. However, the impact of controlling for age distributions was very different between the two years. In 2022 it almost completely eliminated the income differential, while in 2008 it did not change the differential at all.

## Discussion

The study’s main findings were that Arab physicians earn 26% less than Jewish physicians and that this income differential was largely due to differences in the age composition of the two groups (i.e., Arab physicians tended to be younger than Jewish physicians).

There were apparently several reasons why Jewish physicians tended to be older than Arab physicians. One is that the share of Arabs among newly licensed physicians had increased greatly over the prior decade. A second reason was that service in the Israel Defense Forces was (and continues to be) mandatory for all Jews reaching age 18, but not for most Arabs. A third, though probably minor, factor may have been the tendency of Arabs to retire at younger ages than Jews.

The study also found that Arabs were under-represented in supervisory roles, which were associated with substantially higher incomes. Several factors probably contributed to the under-representation of Arab physicians in supervisory roles. A major factor was that a relatively substantial percentage of Arab physicians were under age 40 and that most supervisory roles were occupied by physicians over age 40. But it also the case that among physicians over age 40 Arabs physicians were less likely than Jewish physicians to occupy supervisory roles. It is not clear to what extent this reflected differences in supervisory capabilities and potential, differences in interest in pursuing supervisory roles, and discrimination – either conscious or unconscious.

The study also found that Arab physicians were more likely than Jews to be employed either as generalists or as family physicians. This was probably due in part to the relatively high proportion of Arab physicians who had studied medicine outside of Israel and the relative difficulty that physicians who had studied outside of Israel have in securing residency positions [15]. Another factor may have been a preference among some Arab physicians to practice within the Arab community, leading to family medicine as a common specialty choice.

Will the Arab-Jewish wage differential among physicians increase or decrease in the years ahead? We do not really know. On the one hand, the fact that most of the differential was due to age suggests that the differential could narrow, as more Arab physicians naturally move into the age categories in which physicians earn more. On the other hand, one of the pathways through which age affected income was by increasing the likelihood of moving into a supervisory role, and we do not yet know the extent to which Arab physicians will be filling these roles. This could depend, in part, on the extent to which the health system encourages and prepares young Arab physicians to pursue supervisory roles.

The extent to which young Arab physicians are able to gain entry into the more lucrative specialties will also affect the future of the Arab-Jewish income differential among physicians. This will require both securing relevant residency training slots and passing the relevant specialty certification examinations. Both of these are currently challenging for many Arab physicians who studied in medical schools outside of Israel.

### Independent income v salaried income

Our study found that the raw Arab-Jewish income differential for independent income was approximately triple that for salaried income (53% v 17%). It also found that the full regression model explained much more of the variance for salaried income than for independent income (25% v. 9%). The widespread coverage in collective bargaining agreements of salaried work (but not independent work) is probably a key contributor to both of those differences.

In addition, opportunities for independent work (which is largely done outside the National Health Insurance system) are probably greater in geographic areas with relatively high concentrations of Arab patients and Arab physicians. This is because care outside the NHI system is generally paid for either out-of-pocket or through voluntary private insurance, and Arab patients tend to have lower incomes and less private insurance than Jewish patients. This could be a significant contributor to the finding that, for Arab physicians, independent income was a relatively small proportion of total income.

### Comparison of our study with key studies from the US and the UK

We compared our findings regarding *average* Arab-Jewish differences among physicians of all ages in Israel in 2022 with those of a similar study by Ly et al. regarding *median* Black-White differences among physicians aged 35 + in the United States in 2010–2013 [10]. That study found that in the U.S., after adjusting for age and hours worked, there was a 15% Black-White differential overall, which was much higher among males (26%) than among females (7%).

Further research is needed to understand why minority-majority adjusted income differences were larger in the Ly study regarding the U.S. than in our study regarding Israel.^12^ It could be that part of that difference is due to methodological differences, such as the time period covered^13^, the ages covered, or the choice of summary statistic. There might also be systemic factors related to differences in health systems and/or broader social and economic phenomena. In particular, it may be due to the much greater prevalence of collective bargaining over physician wages in Israel, compared with the U.S.

Two major studies of ethnic pay gaps have been carried out regarding physicians in the National Health Service in England, using median basic pay (i.e. excluding overtime and bonus pay) as the income measure. One of those studies, which did not distinguish among minority groups or between men and women, found – within pay grades - a small pay gap favoring white doctors [18]. Furthermore, it found that the gap between white and BME (Black and Minority Ethnic) physicians was 1% or less for most professional grades, but was 5% for consultants – the highest professional grade. The published article did not indicate the magnitude of the overall white-BME differential (i.e., cutting across pay grades), making it difficult to compare the findings with our own study.

The second NHS study examined gender-ethnicity pay gaps in an intersectoral approach, and also disaggregated the BME group into six different minorities [12]. It found that ”Pay gaps relative to white men vary with the ethnicity-gender combination. Indian men slightly outearn white men and Bangladeshi women have a 40% pay gap. In most cases^14^, pay gaps can largely be explained by characteristics that can be measured, especially grade, with the extent varying by specific ethnicity-gender group. However, a portion of pay gaps cannot be explained by characteristics that can be measured.” The study also found that “All ethnicity-gender groups earned less than white men, except for Indian male doctors who earned slightly more… Bangladeshi men were noticeably the most disadvantaged male category, earning on average approximately 20% less than white men”.

Significantly, neither of the UK studies examined the extent of pay gaps after controlling only for age and sex, innate characteristics which cannot be influenced by the health care system. Instead, they focus on the extent to which there are pay gaps after controlling for professional rank, a characteristic which can be influenced by the health system and which could reflect discrimination.

Note that in the UK, as in Israel, but in contrast to the US, collective bargaining is a major determinant of physician incomes. Thus, it is not surprising that - in the UK - pay grade explains a substantial portion of inter-ethnic pay gaps.

Looking to the future, we encourage our colleagues in other countries to adopt our distinction between inherent demographic characteristics and non-inherent practice characteristics when analyzing the factors contributing to income differences between minority and non-minority physicians.

### Limitations of the current study

The present study has several limitations. One limitation is that the Census database lacks information on several important variables of interest. These include years of experience, specific medical specialty, and perhaps most importantly – place of study. The latter is significant because Arab physicians were much more likely than their Jewish counterparts to have studied medicine outside of Israel. Study within Israel is generally perceived by health care employers as a quality indicator, and it may well impact physician incomes.

Note that while age was an important variable in its own right, it should not be construed as a perfect proxy for experience, particularly in this study. That is because Arabs are not required to serve in the IDF they tend to complete medical school, and begin to accrue experience as physicians, at earlier ages than Jews. At any given age (over age 30), Arab physicians will tend to have several more years of work experience as physicians compared with their Jewish counterparts of the same age. This issue is explored further in Appendix F, which suggests that such an “experience effect” could be non-negligible, but it is not predominant.

Another study limitation is that the Census database (in keeping with the CBS’ classification of occupations) grouped together under the rubric of “generalist physicians” both physicians who lack board certification and physicians who are board-certified in family medicine. These two groups differ substantially in terms of the extent of training they have received, and this is probably reflected in their incomes.

### Avenues for further research

The study findings suggest several promising avenues for further research. One is to assess the extent to which Arab-Jewish income differentials among physicians are related to differences in place of study and specific specializations. This could be explored by linking data from the Ministry of Health’s physician licensure registry. We hypothesize that adjusting for place of study will increase the incomes of Arab physicians relative to Jewish physicians.

Another avenue for further research would be to control for experience. This would require having the Central Bureau of Statistics (CBS) merge census data with longitudinal employer-employee data from the tax authority. However, even then the analysis would have an important limitation: the dataset would not include the work done by physicians independently or for relatively small employers. We hypothesize that adjusting for differences in years of experience would reduce the age-specific incomes of Arab physicians relative to Jewish physicians.

A third avenue for further research can be carried out solely on the basis of the Census data: an examination of the factors contributing to Arab-Jewish income differentials in other professions with substantial numbers of Arab professionals. Such an analysis could both broaden our general understanding of Arab-Jewish income differentials in the professions and clarify, through comparative analysis, whether, how, and why, medicine is unique among these professions.

### Contributions to the literature

This study contributes to the growing body of literature on labor market inequalities by focusing on Arab-Jewish wage differentials in the medical profession. While previous research highlighted Arab underrepresentation in high-paying sectors, this paper offers a detailed examination of income disparities within a high-status profession. The study also utilizes newly available micro-level census data, allowing for a more granular analysis of the factors driving these disparities.

### Policy implications

Preventing income disparities is essential not only for ensuring fairness, but also for fostering a more motivated and effective healthcare workforce. Accordingly, the main study finding, that age-adjusted income disparities between Arab and Jewish physicians are minimal, is encouraging. As such, health care can serve as a model for other industries in Israel where significant income disparities exist between Arab and Jewish professionals.

At the same time, health system leaders cannot rest on their laurels. The situation could erode, due to the recent surge in the number of Arab physicians who studied medicine abroad and the difficulties they are likely to face in securing well-paying positions.

Health system leaders should take steps to promote access of Arab physicians to residency training slots and managerial positions, as well as success in those roles. Most importantly, they should undertake a multi-pronged effort to increase the number of Arabs studying in Israeli medical schools, as Israeli medical school graduates (IMSGs) are more likely than foreign medical school graduates (FMSGs) to compete successfully for such positions.^15^ In fact, among IMSGs the likelihood of securing a residency position within two years of graduation is the same for Arab and Jewish graduates (86%).^16^ (15)

Currently, Israeli Arabs are significantly under-represented in Israeli medical schools.^17^ The main barriers facing Israeli Arabs in gaining acceptance to Israeli medical schools include the overall shortage of slots at such schools, the resulting intense competition for those slots, limited Hebrew language instruction in Arab secondary schools, and inadequate preparation of Arab applicants for medical school interviews and entrance examinations.

The Ministry of Health and the Council for Higher Education have already undertaken a major effort to expand the number and size of Israeli medical schools. But that alone will not be enough to increase Arab representation. It is also important that the Ministry of Education, in cooperation with leaders of Arab society, increase the quantity and quality of Hebrew language instruction in Arab elementary and high schools. The Ministry of Education, together with the Ministry of Health and the Ministry of Social Equality, should develop (or fund) mentorship programs and interview preparation programs for talented Israeli Arab high school students interested in pursuing medical careers. The Council for Higher Education could also provide support for such programs.

Interventions are also needed to support the career development of Israeli Arabs when they are in medical school, residency training, and beyond. These include skill enhancement programs, mentorship programs, and leadership development programs. The Ministry of Health can provide overall leadership on these efforts. Others in a position to advance these interventions include the Israel Medical Association (IMA) and its Scientific Council, the Israel Association of Arab Physicians (an IMA affiliate), and hospital directors and department heads.

## Conclusions

The 2023 Arab-Jewish income differential among physicians was due primarily to differences in age composition. The differential could potentially shrink in the decades ahead, as more Arab physicians reach the ages at which physician incomes are at their highest. However, as part of the effect of age on income is mediated by work characteristics, the extent to which the wage gap will shrink will depend in part on the extent to which Arab physicians will secure prestigious residency training slots and managerial positions. Health system leaders can play an important role in promoting such developments. In particular, steps should be taken to increase the representativeness of Arabs in Israeli medical schools.

## Figures and Tables

**Fig. 7:**
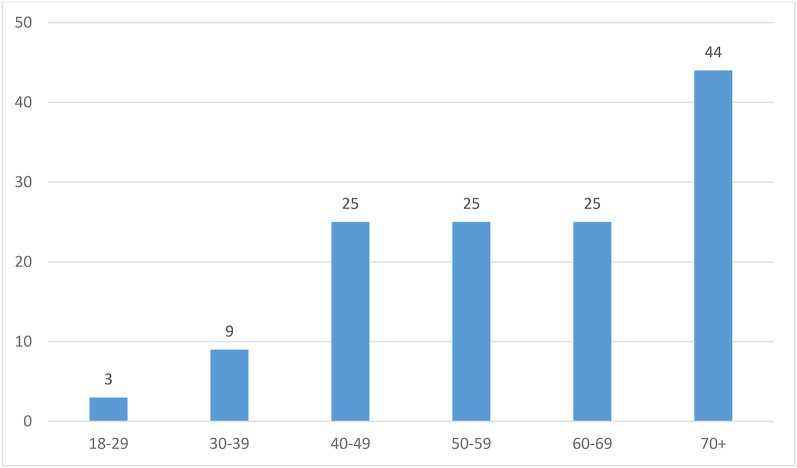
Independent income as a percentage of total income, by age group

**Table 18:**
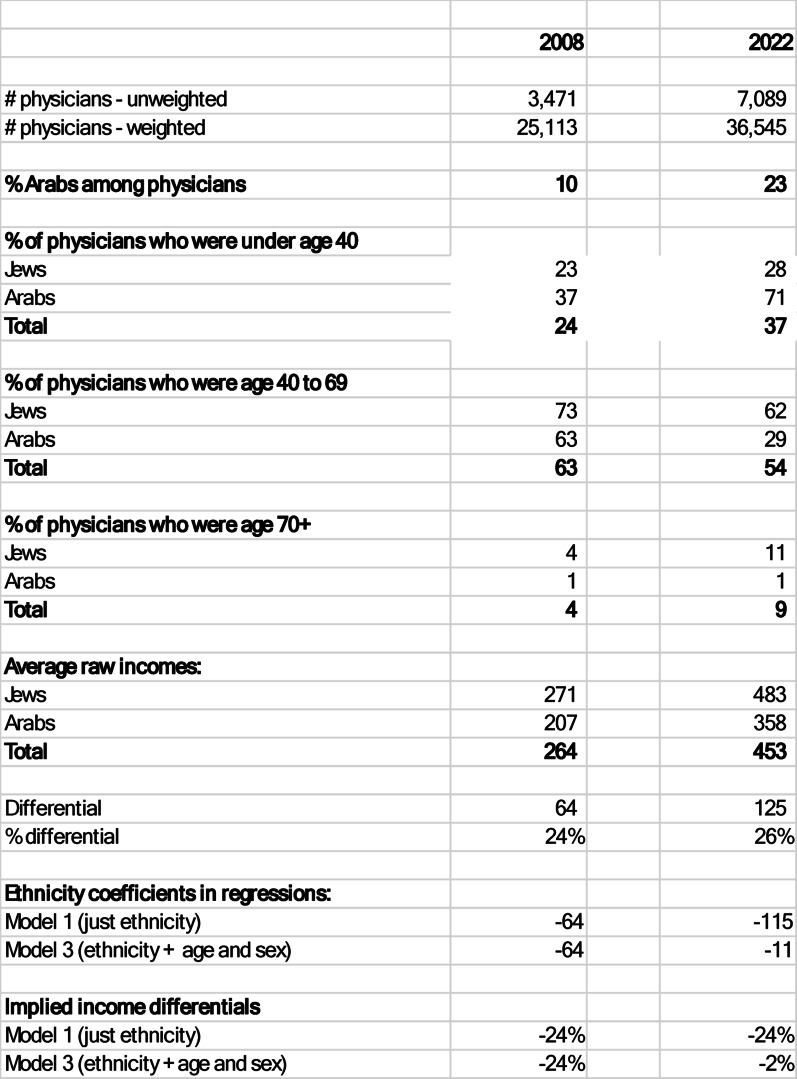
Arab and Jewish physicians in the 2008 and 2002 censuses

**Fig. 8:**
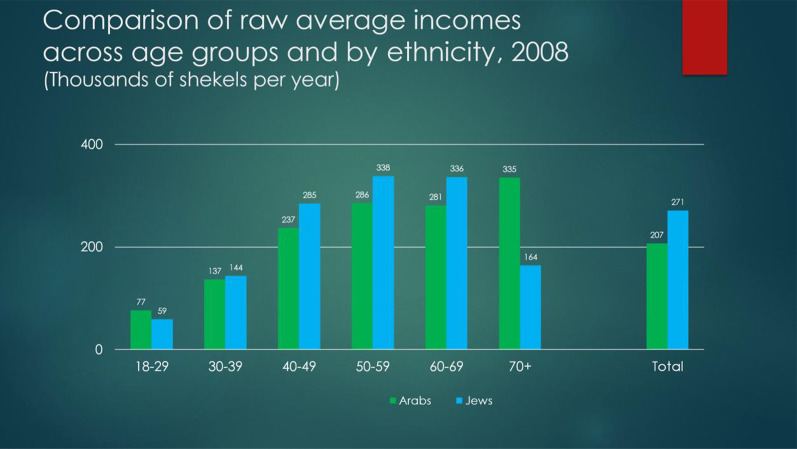
Comparison of raw average incomes across age groups and by ethnicity, 2008

**Fig. 9:**
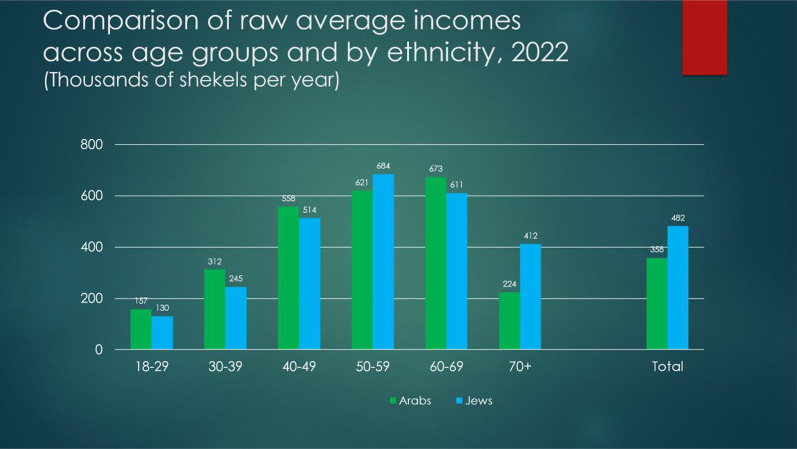
Comparison of raw average incomes across age groups and by ethnicity, 2022

## Data Availability

The authors will be happy to consider requests to share the results of all analyses carried out for this study. However, the raw data are the property of the Israel Central Bureau of Statistics and they are not publicly available.

## Appendix A: Separating out salaried income and independent income

*Background*

Data from the 2022 Census indicate that approximately 93% of Israeli physicians had income as salaried employees, predominantly working for large health care organizations (such as the Ministry of Health, hospitals, and health plans^18^). At the same time, 38% of Israeli physicians had some income from independent work. What makes this possible is that 33% of Israeli physicians had both independent and salaried income.

Israeli physicians receive independent income through several channels, which span community-based and hospital-based care. Regarding community-based care: in two of the health plans, most primary care physicians (PCPs) work predominantly or exclusively as independents, while in a third health plan about half the PCPs work as independents, and in the fourth health plan about a third do so (usually in addition to salaried work for the plan). Independent work is also the predominant mechanism through which all four health plans engage specialists for the care they provide in community settings.

In the hospitals, there is an important distinction between the public hospitals and the private hospitals. In the private hospitals, of which there are only a few in Israel, most physicians work as independents (with residents, pathologists, and radiologists constituting exceptions). In the public hospitals, which constitute the main source of inpatient care in Israel, opportunities for independent work are much more limited. There, even when inpatient care is financed via supplemental or commercial insurance, and the physician is compensated for that care at a higher rate, the hospitals typically pay the physicians for that care through their salaries.

Other sources of independent income include preparation of expert opinions for the courts and freestanding private clinics.

In light of the above, it is important to keep in mind that “independent income” and “private income” are not synonymous. Independent income can be publicly financed as in the case of independent PCPs employed by the health plans. Conversely, salaried physician income can be privately financed as in the case of private financing of care at public hospitals employing salaried physicians.

*Findings regarding all physicians taken together*

Taking all physicians together, income from salary accounted for about three-quarters of total income, while income from work as an independent accounted for about one-quarter total income. Significantly, the percent of income from independent work was about the same for “generalists” and “specialists” – 25% and 23% respectively, while it was negligible for residents (2%). In contrast, this percentage varied substantially across age groups, as indicated in fig. 7. There was also substantial variation across regions, with the lowest percentages found in the North (17%) and in the South (16%).

*Findings comparing Jewish and Arab physicians*

As indicated in Table 6, the percent of income from independent work was 25% for Jewish physicians, while it was 16% for Arab incomes. Moreover, it indicates that the gross Arab-Jewish physician income differential was 26% for total income, 17% for salaried income, and 53% for independent income.

Table 7 compares the expanded model (including demographic, regional, and work variables) for total income, salaried income, and independent income. Note that the coefficient of the “Arab” variable was negative, relatively small and not significant for total income, salaried income, and independent income.

Thus, even for independent income, controlling for demographic, regional and work characteristics reduced the Arab-Jewish income differential from 53% to 2%. It appears that the much lower levels of independent income among Arabs are due to a significant extent to their concentration in the peripheral regions and in the younger age groups. Additional analyses, not displayed here, indicate that the predominant factor is age.

**Table 6:** Average annual physician income by income type and ethnicity (includes those with zero income)

	Total	Arabs	Jews	Differential
Total	454	358	483	−26%
Salaried	349	302	362	−17%
Independent	105	56	120	−53%
Pct independent	23%	16%	25%	

**Table 7:** Regression results by type of income (expanded model)

	Salaried	Independent	Total
R-squared	0.25	0.09	0.31
Constant	237,271	2,263	239,534
Ethnicity (Arab)	−754	−4,581	−5,335
Sex (Female)	**−67**,**059**	**−44**,**766**	**−111**,**825**
Age 30–39	**75**,**385**	6,505	**81**,**890**
Age 40–49	**174**,**366**	**99**,**829**	**274**,**195**
Age 50–59	**276**,**750**	**140**,**853**	**417**,**604**
Age 60–69	**235**,**287**	**123**,**052**	**358**,**339**
Age 70 +	13,453	**146**,**253**	**159**,**707**
Non-supervisor	**−146**,**483**	**−7**,**628**	**−154**,**111**
Resident	**29**,**400**	**−14**,**581**	14,818
Specialist (not FP)	**73**,**348**	**13**,**814**	**87**,**162**
Months worked	**19**,**104**	**5**,**210**	**24**,**286**
North	1,855	**−29**,**879**	**−28**,**024**
Haifa	−3,571	**−21**,**687**	**−25**,**258**
Center	**−42**,**668**	9,197	**−33**,**470**
Tel Aviv	**−43**,**492**	**−8**,**614**	**−52**,**106**
South	**37**,**164**	**−46**,**803**	−9,639
Judea and Samaria	**−30**,**048**	9,451	−20,579

Coefficients in bold are significant at the 0.05 level

Note also that the regression for salaried income explains 25% of the variance, while the regression for independent income explains only 9% of the variance. This is consistent with differences in how incomes are determined for salaried v. independent physicians. For salaried physicians, incomes are largely determined by national collective bargaining agreements, whereas with independent income can vary greatly by locality and other parameters due to differences in supply and demand.

## Appendix B: Findings from the split sample regressions and the regressions using interaction terms

Table 8 displays the results of regressions for the expanded model (model 5) for the full sample as well as split samples of Jews and Arabs. A key finding was that the age coefficient for the 30–39-year-olds was higher for the Arab sample than for the Jewish sample, while the reverse was true for physicians age 70+. Another key finding was that the negative coefficient for females was lower for the Arab sample than for the Jewish sample.

**Table 8:** Regression results for income – expanded model Comparison of split samples and full sample

	All	Jews	Arabs
R-squared	0.31	0.27	0.42
Constant	239,534	229,750	263,364
Ethnicity (Arab)	−5,335	**NA**	**NA**
Sex (Female)	**−111**,**825**	**−123**,**270**	**−57**,**685**
Age 30–39	**81**,**890**	**59**,**528**	**111**,**672**
Age 40–49	**274**,**195**	**254**,**004**	**313**,**856**
Age 50–59	**417**,**604**	**408**,**360**	**365**,**354**
Age 60–69	**358**,**339**	**336**,**056**	**408**,**129**
Age 70 +	**159**,**707**	**145**,**084**	14,838
North	**−28**,**024**	**−36**,**170**	**−20**,**931**
Haifa	**−25**,**258**	**−33**,**793**	**−19**,**661**
Center	**−33**,**470**	**−38**,**578**	**−20**,**020**
Tel Aviv	**−52**,**106**	**−55**,**821**	**−45**,**012**
South	−9,639	−11,206	−22,086
Judea and Samaria	−20,579	−25,767	**NA**
Non-supervisor	**−154**,**111**	**−152**,**852**	**−151**,**898**
Resident	14,818	9,635	**27**,**268**
Specialist (not FP)	87,162	**87**,**167**	**77**,**128**
Months worked	**24**,**286**	**27**,**373**	**18**,**737**

Coefficients in bold are significant at the .05 level

Table 9 displays the regression results for model 3 when interaction terms are included. The coefficient of the Arab-female interaction term was positive and significant. The coefficients of the Arab-age interaction terms were almost all statistically significant, but were variable in including both negative and positive coefficients, without any clear pattern.

**Table 9:** Regression results for total income using the basic model (model 3) with interaction terms added

*N* (weighted)	36,554
R-squared	0.26
Constant	231,285
Ethnicity (Arab)	**−51**,**051**
Sex (Female)	**−139**,**089**
Age 30–39	**90**,**526**
Age 40–49	**356**,**279**
Age 50–59	**522**,**026**
Age 60–69	**440**,**075**
Age 70 +	**218**,**704**
Arab-Female	**66**,**550**
Arab − 30–39	**59**,**598**
Arab − 40–49	32,629
Arab − 50–59	**−72**,**921**
Arab − 60–69	**58**,**605**
Arab 70+	**−175**,**193**

## Appendix C: Comparison of income deciles for Arab and Jewish physicians along with quantile regressions

As indicated in Table 10, the Arab-Jewish income differential differed across income deciles. With an increase in deciles, the differential increased in absolute terms. There was no clear trend in the percentage differences.

**Table 10:** Physician income deciles by ethnicity

	Total	Arabs	Jews	Diff	Pct Diff
10	93	75	101	26	26%
20	165	118	186	68	37%
30	242	184	258	74	29%
40	295	244	314	70	22%
50	353	293	382	89	23%
60	433	331	473	142	30%
70	544	405	582	177	30%
80	695	534	739	205	28%
90	928	732	965	233	24%
N=36,545					

**Table 11:** Quantile & standard regression results for physician incomes, using the basic model (model 3)

	Standard	q = 0.25	q=0.50	q = 0.75
N (weighted)	36,554			
R-squared	0.25	0.12	0.18	0.24
Constant	208,214	80,830	159,416	252,954
Ethnicity (Arab)	−5,413	**19**,**251**	**20**,**228**	**16**,**280**
Sex (Female)	**−126**,**857**	**−49**,**607**	**−84**,**733**	**−112**,**466**
Age 30–39	**119**,**073**	**96**,**025**	**129**,**796**	**137**,**466**
Age 40–49	**373**,**128**	**240**,**122**	**345**,**346**	**475**,**951**
Age 50–59	**527**,**712**	**327**,**994**	**475**,**644**	**677**,**863**
Age 60–69	**460**,**549**	**279**,**710**	**422**,**706**	**625**,**977**
Age 70 +	**230**,**736**	**98**,**280**	**190**,**989**	**284**,**577**

Coefficients in bold are significant at the .05 level

Table 11 presents the results of quantile regressions, in comparison with the standard regression, for model 3. In the quantile regressions the coefficient of the ethnicity variable was positive and significant, whereas in the standard regression it was negative and non-significant. This apparently reflects the concentration of Arab physicians in the lower income quantiles, along with an Arab advantage within quantiles.

## Appendix D: Findings from the regressions with log of income as the dependent variable

Economists often use the natural log of income (instead of income, per se) as the dependent variable in regression analyses. Such models are popular among economists because in wage regressions the assumption of a constant returns (i.e., a constant effect in percentages, or semi-elasticities) is often more reasonable than that of a constant marginal effect (in levels - shekels). When using these models, the exponentials of the coefficients indicate the independent multiplicative effect of the relevant variables (i.e. the approximate percentage changes).

Table 12 presents the findings for models 1, 3, and 5. The pattern (in terms of which coefficients are statistically significant) was pretty much the same for the regressions using income (Table 4). Table 13 indicates that, prior to controlling for other variables, Arabs earned on average 29% less than Jews, but after controlling for age and sex they earned 4% more than Jews. Controlling for region and practice characteristics increased this to 7%.

**Table 12:** Results for regressions using natural log of income (before exponentiation)

	Model 1	Model 3	Model 5
R-squared	0.03	0.32	0.43
Constant	12.79	11.73	10.73
Ethnicity (Arab)	**−0.34**	**0.04**	**0.07**
Sex (Female)		**−0.29**	**−0.23**
Age 30–39		**0.68**	**0.50**
Age 40–49		**1.38**	**1.02**
Age 50–59		**1.63**	**1.23**
Age 60–69		**1.50**	**1.13**
Age 70 +		**0.93**	**0.67**
North			**−0.03**
Haifa			**−0.06**
Central			**−0.03**
Tel Aviv			**−0.06**
South			**0.07**
Judea and Samaria			0.01
Non-supervisor			**−0.27**
Resident			−0.01
Specialist (not FP)			**0.20**
Months worked			**0.14**

Coefficients in bold are significant at the .05 level

**Table 13:** Coefficients after applying exponentiation

	Model 1	Model 3	Model 5
R-squared	0.03	0.32	0.43
Constant	358,613	124,244	45,707
Ethnicity (Arab)	0.71	1.04	1.07
Sex (Female)		0.75	0.79
Age 30–39		1.97	1.65
Age 40–49		3.97	2.77
Age 50–59		5.10	3.42
Age 60–69		4.48	3.10
Age 70 +		2.53	1.95
North			0.97
Haifa			0.94
Central			0.97
Tel Aviv			0.94
South			1.07
Judea and Samaria			1.01
Non-supervisor			0.76
Resident			0.99
Specialist (not FP)			1.22
Months worked			1.15

## Appendix E: Conjoint consideration of ethnicity and sex

As can be seen in Table 14, the Arab-Jewish income differential among physicians was larger for females than for males (39% v. 30%). Interestingly, both of these percentages were higher than the 26% overall differential when both sexes are taken together. This somewhat anomalous pattern was due to the fact that males) who tend to earn more than females) were much more prevalent among Arab physicians than among Jewish physicians (77% v. 52%).

**Table 14:** Average physician incomes by ethnicity and sex

	Jews	Arabs	Total	Differential
Males	558	392	507	30%
Females	401	243	382	39%
Total	483	358	454	26%
Differential	28%	38%	25%	

N = 36,545

Table 14 also indicates that the male-female income differential was higher among Arab physicians than among Jewish physicians (38% v. 28%). This was probably due, at least in part, to differences in age distribution (Table 15). 86% of the Arab female physicians were under age 40, compared with 66% for Arab males, 32% for Jewish females and 23% for Jewish males. This explanation is supported by the regressions displayed in Table 9 in which, after controlling for age, the Arab-female term had a positive and statistically significant coefficient.

The concentration of Arab female physicians in the younger age groups probably also contributed to the first finding noted in this section – that the Arab-Jewish income differential is larger for females than for males.

The concentration of Arab female physicians in the younger age groups was probably due largely to a recent expansion in the number of newly licensed female Arab physicians.

Our finding that in both Israeli ethnic groups female physicians earn less than male physicians is consistent with findings from the US and the UK (10–12). In contrast, while we found that the Arab-Jewish income gap was greater among females than among male, in the US study the Black-White income gap was greater among males than among females (10). Further research is needed to explore the reasons for this difference between Israel and the US.

**Table 15:** Percent of physicians under age 40, by ethnicity and sex

	Jews	Arabs	Total
Males	23	66	36
Females	32	86	39
Total	27	70	37

## Appendix F: Analyses using adjusted age

The study found that, controlling for the inherent demographic factors of sex and age, reduces the Arab-Jewish income differential among Israeli physicians from 26% to 1%. One of the possible explanations for the almost total lack of a sex/age-adjusted differential is that at any given age Arab physicians have more experience on average than Jewish physicians.

Unfortunately, we do not have data comparing Arabs and Jews regarding the average years of experience, by age. As a sensitivity analysis, we consider the case in which, at any given age, Arabs have three more years of experience than Jews. We chose three years, as this is the round number closest to the standard amount of “regular service” time served by male^19^ IDF conscripts (2 years and 8 moths). While IDF service is required of all Israeli Jews, it is not required for most Israeli Arabs.^20^ This was operationalized through a new variable called “adjusted age” which was set equal to age in the case of Jews and equal to age + 3 in the case of Arabs.

As can be seen in Table 16, when controlling for sex and adjusted age the coefficient of the ethnicity variable was − 41,236, which was approximately 8% of the average Jewish physician income. This is larger than the differential found when adjusting for unadjusted age (1%). Still, controlling for sex and adjusted age reduced the raw Arab-Jewish income differential by more than two-thirds – from 26% to 8%. This suggests that if Arab physicians indeed have on average 3 more years of experience than their Jewish counterparts of the same age, then this additional experience translates into a 7% enhancement of total income.^21^

**Table 16:** Comparing the Model 3 regression results for total income, when using adjusted age instead of actual age

	**Actual**	**Adjusted**
	**Age**	**Age**
R-squared	0.25	0.25
Constant	208,214	203,607
Ethnicity (Arab)	-5,413	**-41,236**

Coefficients in bold are significant at the 0.05 level

## Appendix G: Arab-Jewish income differentials for selected professions

Table 17 compares physicians with other professions with substantial Arab representation, regarding Arab employment shares and overall average incomes, as well as the average Arab-Jewish income differentials in percentage terms. The percent income differentials are presented both for raw income and income adjusted for age and sex. The professions are ordered in terms of the raw percent Arab-Jewish income differential. The table indicates that physician incomes were higher than for all the other professions included in the table and that the Arab share in employment for physicians was intermediate. It also indicates that – for physicians - the raw Arab-Jewish income differential, in percentage terms, was substantially higher than it was for nurses and teachers, and substantially lower than it was for dentists and lawyers.

In addition, the table shows that adjustments for age and sex greatly reduced the income differential in medicine and nursing, but not in the other professions examined. After adjusting for age and sex, Jews earned only a bit more than Arabs in medicine and nursing, much more than Arabs in dentistry and law, and less than Arabs in teaching.

**Table 17:** Average incomes, average Arab-Jewish income differentials and Arab employment shares in selected professions

	Arab employment shares(percents)	Average incomes (thousands of NIS)	Raw Arab-Jewish income differentials (percents)	Adjusted Arab-Jewish income differentials (percents)
Dentists	33	263	50	54
Lawyers	10	251	45	46
**Physicians**	**23**	**453**	**26**	**2**
Nurses	26	190	10	3
Pharmacists	50	186	9	9
Teachers	21	139	-7	-10

## Appendix H: Comparison of key findings for 2008 and 2002

Prior to 2022, the most recent CBS census was in 2008. Table 18 compares the findings from the two censuses on parameters that are central to this manuscript.

As indicated in the table, in 2008 only 10% of the physicians were Arabs, well below the 23% figure for 2022. Moreover, while in both years Arab physicians were younger than their Jewish counterparts, the differences in the age mix were much more pronounced in 2022.

In both years, the raw income differential between Arab and Jewish physicians was approximately 25%. However, the impact of controlling for age distributions was very different between the two years. In 2022 it almost completely eliminated the income differential, while in 2008 it did not change the differential at all.

As can be seen by comparing Figures 8 and 9, the pattern of age-specific income differentials changed between 2008 and 2022. In 2008, Jewish physicians earned more than Arab physicians except in the under 30 age group and in the over 70 age group (which included only 4% of the Jewish physicians and only 1% of the Arab physicians). In 2022, if four of the six age groups examined, Arab physicians earned more than the Jewish physicians.

Thus, in 2008, the overall Arab-Jewish income differential for physicians was due predominantly to differences in age-specific incomes. In contrast, 2022 the differential was due predominantly to differences in the age distributions.

Further research is needed to understand why the age-specific income differentials narrowed between 2008 and 2022.

## Footnotes

1 The increase in the percentage of physicians who are Arabs was clearly quite rapid. A major contributor was the retirement of a very large number of Jewish physicians during this period. Many of them were Jewish physicians who had arrived in Israel circa 1990 as part of the massive immigration of Jews from the Former Soviet Union.

2 While some of the Arab medical students may have studied outside of Israel because they prefer to study in another country, the primary reason for most of them is probably difficulty in being accepted to an Israeli medical school. The entry requirements for Israeli medical schools are extremely high and require outstanding scores on both matriculation exams taken during high school and on an independent psychometric exam. Arab students are at a disadvantage because, on average, Arab secondary schools are more poorly financed and less well staffed than Jewish secondary schools. In addition, Arab students are less likely to be able to afford private preparatory courses for the psychometric exams.

3 Thus, in the raw sample, Arabs constituted 19% of the physicians. After weighting, this figure increased to 23%.

4 In the appendices we present supplementary analyses in which the dependent variables were gross annual income from salaried employment, gross annual income from independent work, and the natural logarithm of total gross annual income.

5 Almost all the Jews residing in Judea and Samaria (i.e. the West Bank) are citizens of the State of Israel, and hence they are included in the CBS Census data.

6 The differential for *median* physician incomes was slightly lower, at 23%.

7 Incomes were highest in Judea/Samaria (the West Bank); note that CBS data include only Jews residing in that area.

8 The North was also characterized by a very high concentration of Arabs in general, irrespective of profession.

9 As indicated in Appendix A, the North was also characterized by a relatively low percentage of income coming from independent work.

10 In a regression in which age was included but sex was not included (not shown in the table), the R-squared was 0.23 and the coefficient of the Arab indicator variable was + 37,485 (i.e. a differential in favor of the Arab physicians). The addition of the sex variable decreased the coefficient of the Arab indicator variable from 37,485 to -5,413. This makes sense as male physicians tend to earn more than female physicians and Arab physicians are more likely to be male than are Jewish physicians.

11 This was due primarily to Arab physicians being markedly younger than Jewish physicians.

12 Another difference between the countries is that in the United States Blacks and Hispanics are significantly underrepresented in medicine and other health care professions [16, 17] while this is not the case for Arabs in Israel [13].

13 A 2022 publication by Ly [16] concludes that “income differences between Black and White male physicians have only modestly improved since 1960 and remain substantial”. Still, from a chart in that article, it appears that - in percentage terms - the differential narrowed somewhat between 2010 and 2018.

14 Black men constitute the main exception; in their case observed characteristics do not account for most of the pay gap.

15 There are many reasons why directors of residency programs prefer IMSGs over FMSGs. These include the IMSGs’ greater familiarity with the Israeli health care system, their better Hebrew language skills, and their better training in clinical skills.

16 This, despite the substantial discretion that department heads have in choosing among applicants for residency positions. While that discretion is believed to be a barrier for FMSGs, it does not appear to be a barrier for IMSGs.

17 This contrasts with the situation in foreign medical schools, where Israeli Arabs are over-represented.
